# Effects of Pokémon GO on Physical Activity and Psychological and Social Outcomes: A Systematic Review

**DOI:** 10.3390/jcm10091860

**Published:** 2021-04-25

**Authors:** Jung Eun Lee, Nan Zeng, Yoonsin Oh, Daehyoung Lee, Zan Gao

**Affiliations:** 1 Department of Applied Human Sciences, University of Minnesota-Duluth, Duluth, MN 55803, USA; lee03284@d.umn.edu; 2 Prevention Research Center, Department of Pediatrics, University of New Mexico Health Sciences Center, Albuquerque, NM 87131, USA; NZeng@salud.unm.edu; 3 Department of Kinesiology, University of Wisconsin-Eau Claire, Eau Claire, WI 54702, USA; yoonsin@uwec.edu; 4 School of Kinesiology, University of Minnesota-Twin Cities, Minneapolis, MN 55455, USA

**Keywords:** augmented reality games, augmented virtual reality, social interaction, mobile augmented games, psychological wellbeing

## Abstract

Augmented reality (AR) mobile game, Pokémon GO, leverages gamification and location tracking technology to encourage players to walk in different places to catch Pokémon characters in real-world settings. The systematic review sought to explore the impact Pokémon GO has on players’ physical activity (PA), and psychological and social outcomes. Six research databases (PubMed, SPORTDiscus, PsycInfo, Web of Science, Science Direct, and Scopus) were used. Study inclusion criteria were: (1) quantitative research published in English; (2) examined the relationships between or impact of Pokémon GO on PA, psychological, and/or social outcomes; and (3) included participants played or exposed to Pokémon GO. Thirty-six studies were included with a total sample of 38,724 participants. Players had significantly greater PA than non-players in terms of daily steps and number of days spent in moderate PA. Pokémon GO game also improved players’ social interactions and their mood/affects. Selective attention and concentration improved in adolescents and memory improved in young adults after playing the game. Findings suggest playing Pokémon GO could promote meaningful improvements in walking behavior, as well as psychological and social well-being. More multidimensional research with randomized controlled trial design is needed to identify factors that influence adoption and sustainability of Pokémon GO playing.

## 1. Introduction

Insufficient physical activity (PA) and increased sedentary behavior are leading risk factors for major chronic diseases, such as diabetes, cardiovascular diseases, hypertension, and depression [1]. While much effort has been put on increasing the awareness of the importance of regular PA, nearly 80% of Americans are not sufficiently physically active [2]. Thus, technology-savvy strategies, such as exergaming and mobile app-based interventions, have been widely used to increase PA enjoyment and participation. Incorporating videogames into exercise, known as exergaming, has been proven promising in promoting PA among various populations [3,4,5,6]. For example, researchers found some exergames (e.g., Dance Dance Revolution, Wii Fit, and Kinect Sports) were well received and could increase individuals’ PA and psychosocial outcomes, such as self-efficacy, perceived social support, and enjoyment [7].

Augmented reality (AR) games are among the newest technologies available to facilitate the promotion of PA and health. Specifically, AR games combine virtual and real world into one interface to offer players real-life environments where sensory stimuli are integrated with GPS location and graphics [8]. Likewise, AR mobile games involve the integration of this sensory content into game players’ environments that can be displayed on smartphone screens by the cameras [9]. Location-based AR mobile games differ from exergaming designed to promote PA mostly indoor; however, these AR games still demonstrate the potential to promote PA comparable to the recommended levels [10]. Further, AR mobile games might exert positive effects on individuals’ overall well-being as AR games predominately require the players to have some form of movement by combining gaming, walking, and exploring outdoor environments [11,12].

Pokémon GO (Niantic, Inc., San Francisco, CA) AR game, specifically designed for use on mobile devices, reached unprecedented popularity since its international release in July 2016 [13]. The game uses the mobile device’s GPS to locate, capture, and train virtual cartoon characters called Pokémon. The nature of the game not only encourages PA and decreases sedentary behavior as the players maneuver through the play sites, but it also enhances social interaction as nearby players compete with one another or when they play with their friends [14]. Additionally, more evidence has reported the game’s influence on individuals’ psychological and social well-being with increased game plays [15]. Nevertheless, scientific inquiry into the effects of Pokémon GO game play is in its infancy.

Despite AR gaming popularity and the potential to promote PA in various populations, and bring people outdoors, it has not been systematically used as a health promotion tool. Thus, it is essential to examine its potential for PA and health promotion interventions in the communities. Yet, only a few review articles examined the impact of Pokémon GO. One extensive systematic review [16] explored the health effects of Pokémon GO, yet did not include quality assessment of studies included, and the other reviews [17,18] only examined the impact of Pokémon GO on PA. There are a lack of reviews on the impacts of AR gaming on multi-dimensional outcomes, such as psychological and social outcomes beyond PA. In response, the purpose of this review was to systematically examine the effects of Pokémon GO play compared to non-players on individuals’ PA, and psychological and social outcomes in cross-sectional, longitudinal, and experimental studies.

## 2. Materials and Methods

The Preferred Reporting Items for Systematic Review and Meta-Analysis Protocols (PRISMA-P) 2015 statement was consulted and provided the framework for this review [19].

### 2.1. Eligibility Criteria

The following inclusion criteria were used for each study: (1) published in English as peer-reviewed empirical research between July 2016 and April 2021; (2) conducted quantitative research; (3) examined the relationships between or impact of Pokémon GO on any of the following outcomes: PA, psychological, and social outcomes; and (4) included participants played or exposed to Pokémon GO. Studies were excluded if the study (1) conducted qualitative research; (2) focused only on predictors, such as motivation, personality trait, and perception of Pokémon GO as primary outcomes; (3) focused on other healthy behaviors or conditions; or (4) used other AR games.

### 2.2. Data Sources and Search Strategies

Comprehensive and extensive electronic search of English language databases was conducted by consulting the recent published systematic review on Pokémon GO [18] and input from coauthors. The following databases were used for the search: PubMed, SPORTDiscus, PsycINFO, Web of Science, Science Direct, and Scopus. Keywords were used to search for studies of the impact of Pokémon GO on individuals’ PA, psychological, or social well-beings. Sources were searched with the key terms and used in combination: “Pokémon GO” AND “physical activity” OR “psychosocial” “psychological” “depression” OR “anxiety” OR “affect” OR “mood” OR “social health” OR “social relation” OR “mental health” OR “cognition” OR “cognitive” OR “memory” OR “executive functions”. Relevant studies were further identified by means of cross-referencing the bibliographies of selected articles.

### 2.3. Data Extraction and Synthesis

After the first author (J.E.L.) located the titles and abstracts of potentially relevant articles through keyword search, two researchers (J.E.L. and N.Z.) independently screened the list of titles and abstracts for eligibility. Finally, all potential articles were downloaded as full text, after which two authors (J.E.L. and N.Z.) reviewed each article separately to ensure that only relevant entries were included. Any discrepancies in the determination of the relevance of an article to the topic were discussed and resolved by consensus. When there were difficulties reaching consensus, another co-author (Z.G.) was consulted for a final decision. The following information were extracted from each study on a shared Excel sheet by the first author (J.E.L.) and shared with other researchers (N.Z. and Z.G.) for review: year of publication and country of origin, study design, sample size, and characteristics, such as age and percentage of sex, type of outcomes and instruments used, duration of Pokémon GO play, and key findings on the effectiveness of Pokémon GO on PA, psychological and social outcomes.

### 2.4. Study Quality and Risk of Bias in Individual Studies

The risk of bias in each study was evaluated by two independent authors (J.E.L. and N.Z.) based on the National Collaborating Centre for Methods and Tools: quality assessment tool for quantitative studies [20]. This quality assessment tool included evaluation of the following components: (1) selection bias, (2) study design, (3) confounders, (4) blinding, (5) data collection methods, and (6) withdrawals and dropouts. For each study, global rating was calculated based on the following basis for each component; strong = 1, moderate = 2, and weak = 3. A global rating of strong corresponded to no component rated as weak; a global rating of moderate—if only one weak component; and a global rating of weak included two or more weak components. The basis for rating of each component is described in the note section of Appendix A Table A1. Two authors (J.L. and N.Z.) rated the initial 13 studies to reach good interrater reliability (ICC > 0.80), and then split the remaining articles for individual rating.

## 3. Results

### 3.1. Study Selection

**Figure 1:**
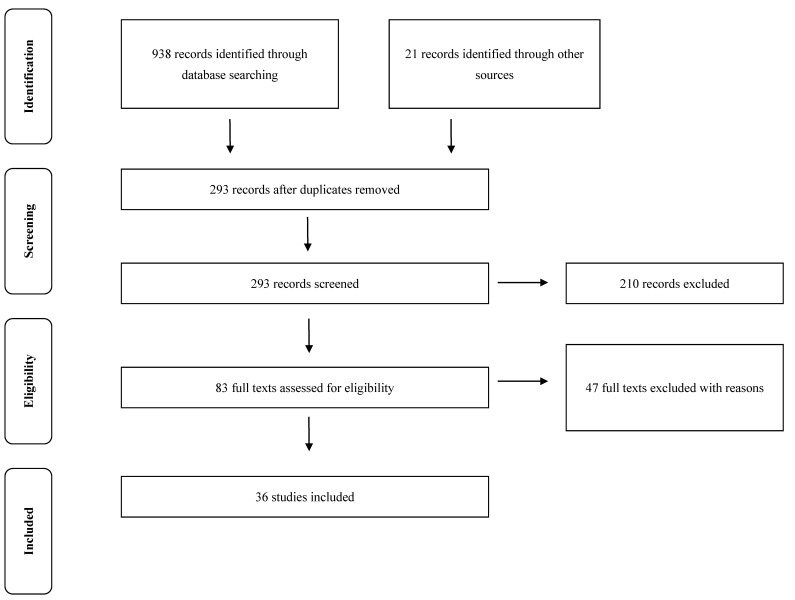
Preferred reporting items for systematic reviews and meta-analyses flow diagram of study selection.

Figure 1 illustrates the flow chart of screening and selection process for the articles. A total of 959 articles were initially identified, 87 from PubMed, 154 from SPORTDiscus, 93 from PsycInfo, 220 from Web of Science, 74 from Science Direct, 310 from Scopus, and 21 from other sources. A total of 293 remained after removing duplicates, and 83 after screening of the titles and abstracts. The remaining 83 full-text articles were further screened according to the inclusion criteria. Of these 83 studies, 47 were excluded for the following reasons: written in non-English language, no outcomes of interests, included other AR game, implemented only qualitative analysis, or publishing two different articles with same sample. In this case, only the parent article was included. Finally, 36 articles [21,22,23,24,25,26,27,28,29,30,31,32,33,34,35,36,37,38,39,40,41,42,43,44,45,46,47,48,49,50,51,52,53,54,55,56] were included for this review.

### 3.2. Study Quality and Risk of Bias Assessment

The analysis of study design quality and the risk of bias are presented in Appendix A Table A1. For the initial ratings of the 70-item scores, the percentage agreement was 92.7%. Among 36 studies, 2 studies received a global rating of strong quality, 11 studies received a global rating of moderate quality, and 23 studies received a global rating of weak quality. The most common issues with the study quality were related to study design and blinding. More specifically, 18 studies ranked weak in the study design, and only one study was rated as strong on blinding across all 36 studies. Other concerns in the study quality included failure to report of dropout rate and control for confounders. Due to variances in study design (quasi-experimental vs. observational study), study sample (i.e., children vs. adults), and study quality (most are rated as high risk); the research team was unable to make a meaningful analysis of pooled data, rendering a meta-analysis impractical.

### 3.3. Study Characteristics

The sample characteristics of the studies are summarized in Table 1. The included articles were published between 2016 and 2020, with most of the articles published in 2018 (*n* = 12) and 2019 (*n* = 10), indicating research in this field is in its infancy, yet expanding. Among the 36 studies, 33 were observational studies, either cross-sectional or longitudinal study, and three studies employed experimental design, yet none was randomized control trial. The study locations varied as 21 studies were conducted in the U.S., 7 studies in Asia (3 studies in Hong Kong, 2 studies in Japan, and 1 study each in Thailand and Taiwan), 7 studies in Europe (2 studies each in Spain and UK, 1 study each in Serbia and Poland), and 1 study in Costa Rica. The length of exposure to Pokémon GO ranged from 60 min to 9 months, with the median intervention length being 1 month.

Across studies, the sample sizes varied from 20 to 27,126. Seven studies included less than 50 participants, 11 studies had a sample size between 50 and 300, and the remaining studies (*n* = 18) had larger than 300 participants. Specifically, 14 studies had sample sizes ranging from 301 to 999, and one study had over 10,000 participants. The total number of participants in all the included studies was 38,724. The age of participants ranged from children (5 years old) to the elderly (over 60 years old). Three studies [31,38,40] included children and adolescents (with the mean age of 13 years), while the rest of the other studies exclusively included adults aged 18 years and older. The percentage of females in the samples ranged from 25 to 78% across the studies.

### 3.4. Outcomes and Instruments

Outcomes and measurements for PA, psychological and social domains are illustrated in Table 1. The measurement methods were valid and used reliable scales and questionnaires. In these articles, the indicators of PA included daily step counts, time spent and/or distance travelled in various activities (i.e., walking, biking, running/jogging, and skating), time spent in sedentary, light, moderate-to-vigorous, vigorous PA, energy expenditure, and exercise frequency. In 14 studies [25,26,29,32,34,36,38,40,43,45,47,50,52,53], PA was assessed solely with subjective measures such as the International Physical Activity Questionnaire. Eight studies [21,24,28,30,33,35,39,41], incorporated only objective measures to assess participants’ PA such as pedometers, accelerometers, wrist or arm-worn activity sensors, heart rate monitors, and mobile applications, such as the iPhone Health app, step-counting app, and ecological momentary assessment app. Finally, five studies [23,37,46,48,49] incorporated both subjective and objective assessments of PA, in which most of the objective measures used health or step-counting mobile applications.

The psychological outcomes varied and included affect [21,44,51], life satisfaction [27,51], emotional intelligence [31,40], vitality and empathy [27], attitude toward PA [29], psychological distress and wellbeing [42,55], neuroticism [56] working memory [21,31,40], selective attention and concentration capacity [31,40], and other cognitive performance, such as mathematical calculation and reading ability [31,40], and creative imagination [31]. Social indicators included interpersonal interaction [27,31,32,40,44,51,52], community belonging [54], empathy, [21] social anxiety, depression, and loneliness [51].

### 3.5. Pokémon GO Effects on Physical Activity

Of the 27 studies that explored the role of Pokémon GO on players’ PA, 19 studies observed Pokémon GO to have positive effects on players’ PA. In terms of comparing the game effects between players and non-players, 7 studies among a total of 12 reported that players had significantly greater PA than non-players in terms of daily steps [22,30,49], number of days per week spent in moderate PA (MPA) and walking [25], and distance walked (km/day) [39,40,48]. For example, Althoff et al. [22] reported that the players increased daily steps by 192 while the comparison group decreased by 50 daily steps. Similarly, Broom and Flint [25] reported that Pokémon GO players demonstrated higher number of days that were spent engaging in MPA and walking compared to non-players. However, in their study [25], there was no significant difference in other physical outcomes, such as time spent in moderate-to-vigorous PA (MVPA) or sitting on weekdays. Additionally, another study [30] found that players maintained their steps counts while non-players showed decline in the winter. When comparing before and after the Pokémon GO use, 11 studies out of 14 indicated that Pokémon GO significantly increased players’ PA as measured by times spent in walking (min/day) [23,36], other types of PA, including walking, running, biking, and skating (hours/day) [26,32], mild and moderate PA (min/week) [28,38], and MVPA (min/day) [50], distance of daily walking and running [35], daily step counts [37] exercise frequency [34], and percentage of days spent more than 10,000 steps [46] after a certain period of the game play.

Despite the positive correlations between game play and PA levels, quite a few raised questions on the sustainability of such effects [22,23]. Although an increasing trend of PA was observed immediately after the download of the Pokémon GO apps or at the beginning of game play, some studies reported that this pattern attenuated after 1–4 weeks of initial download [22,23]. Additionally, some studies indicated that the differences in PA between the players and non-players were not significant beyond week 1 [48]; the average steps went back to pre-installation levels by the sixth week after installation [49]. However, another researcher [30] reported that players’ step counts were significantly higher even 7 months after the release of the game, which was not observed in previous short-term studies.

### 3.6. Psychological and Social Effects of Pokémon GO

Comparing to studies focused on the effects on PA, there were relatively fewer articles that examined psychological and/or social effects (13 out of 36 studies) of Pokémon GO: seven studies investigated both psychological and social outcomes [21,27,31,32,40,44,51], four studies examined only psychological outcomes [29,42,55,56], and two explored only social effects [52,54] of the game. Eleven articles, which indicated psychological benefits of playing Pokémon GO, reported that the use of game was effective in improving affect and mood [21,32,42,51,55,56]. Specifically, it reduced: negative affect after just about an hour of play [21]; neurotic personality trait after 3 months of game play [56]; and psychological distress compared to non-players [42]. Additionally, playing Pokémon GO was related to psychological wellbeing, such as higher life satisfaction, happiness, and vitality [27,44], and various cognitive outcomes, such as verbal working memory in young adults [21], and selective attention, creativity, and concentration levels among adolescents [31,40].

Multiple studies [21,27,31,32,40,44,51,52,54] reported improved social wellbeing after the use of Pokémon GO. It was associated with improvement in emotions crucial in building health interpersonal relationships, such as empathy [21], social anxiety, and depression [32,51]. For example, one study reported reduced anxiety of leaving the house and interacting with strangers after a week of game play [32]. The game play was also beneficial in increased friendship formation and intensification [51], greater social interaction [27], sociability [31,40,44,52], and sense of belonging and connection [54].

## 4. Discussion

Designing innovative and enjoyable PA interventions to promote multi-dimensional well-beings has become crucial as the number of people with obesity and chronic illnesses are increasing in the U.S. [57]. One of the appealing aspects of Pokémon GO is its entertaining and explorative features. Unlike traditional sedentary video games, Pokémon GO requires players to travel to a physical location with unique features that encourage social interactions among friends and other players [57]. This review sought to synthesize and review available literature exploring the effects of Pokémon GO on players’ PA and psychological and social outcomes. Thirty-six studies were included in this review, with 27 studies examining on PA using direct and indirect measurements, 11 studies on psychological outcomes, and 9 studies on social outcomes.

### 4.1. Effects of Pokémon GO on Physical Activity Behaviors

Examining the effects of Pokémon GO on PA was the most prevalent topic in AR game literature. Overall, 18 of 27 studies (66%) showed that either compared to non-players or to before the game exposure, the players were more likely to be active, as measured by steps counts, time spent in walking, light intensity PA (LPA). However, the findings on sedentary behavior were mixed [23,24,25]. Although players have increased their steps through the games, their intensity of PA might fall just short of the ideal quality. Specifically, researchers reported that, although players engaged in more days of MPA and walking, they had fewer vigorous PA days compared to non-players [25]. Moreover, no differences were evident in minutes of MVPA or walking between the groups. In these findings, researchers suggested Pokémon GO games may have the potential issue of replacing MVPA with LPA in young adults. Additionally, in a study that assessed PA levels during a 2-h game play, the researchers found that players actually spent more time in sedentary and LPA, and less time in MVPA than non-players [24]. First, brisk walking is considered MVPA where strolling is considered LPA. The game concept of walking with Pokémon already excludes the potential for players to engage in vigorous PA. Another potential reason might be some players take different modes of transportation to earn distances. Players could play Pokémon GO games any way they want, with different intentions and motives. If they focused on earning game items and leveling their Pokémon by visiting many different virtual gyms (locations) in a short amount of the time, they could care less on being physically active by walking to the places and travel faster with different mode of transportation. For example, some players were reported to drive or take public transportation to different locations, especially those players whose motives for playing the game was competition rather than being more physically active [9]. It seems that, although the game has potential to promote PA, it may not be sufficient to reap the health benefits recommended by the PA guidelines. Therefore, concerted efforts are called to allow this creative tool to supplement traditional exercises, which may encourage players to engage in higher PA intensity.

It is also noteworthy that, despite the increase in participants’ step counts upon initiation of the game play, its long-term effect is questionable, as initial increase in PA levels wane after 3 to 4 weeks into playing the game [49]. Due to the waning effects of Pokémon GO in a short period of time, promoting PA and health behaviors through AR games may be a challenge for those who lose interest in short-term periods [58]. In fact, this novelty issue may be a shared concern for the majority of digital gamified PA interventions [59,60]. However, some literature examining motivation for continuation and discontinuation of Pokémon GO has shed insight on how to improve the game to accommodate the novelty issue. To begin with, researchers found the main motivation for adoption and continuation of the Pokémon GO game was to fulfil needs for mastery experience, social interaction, enjoyment, or competition [37,61]. For example, players usually dropped out of the due to the repetitive nature of the gameplay after catching most of the Pokémon or friends dropping out from the game [9]. Therefore, some of efforts and attention could be directed to raising competency levels and sustaining social features with this game [62]. Specifically, to raise competency levels, researchers [9] suggested that game content could be enhanced with more varieties so that players do not feel it is repetitive within a couple of weeks of playing the game. Video game players naturally form their affinity groups with shared identifies, goals, and practices [63]. The concept of forming affinity groups can present social groups; this will allow players to socialize, exchange tips, and game information, and allow input on others’ posts, as this might prevent novice players from quitting the game [9].

Despite disappointing results on long-term adherence, Pokémon GO has succeeded in its ability to increase PA level among obese and inactive people who were at a higher risk of developing non-communicable disease [58]. Inactive people are encouraged to participate in regular PA and the game seems to enable at least short-term effects in this population. The overweight and obese populations usually set weight loss as their exercise goals, and they are prone to easily lose motivation due to slow progress in achieving the goal. In addition, Pokémon GO seems to be a viable solution as it offers instant gratification during and after game play, including enjoyment through gaming, and other psychosocial benefits.

### 4.2. Psychological and Social Effects of Pokémon GO

Most included studies suggested that playing Pokémon GO was associated with better bond of relationships and sense of emotional well-being [21,27,31,32,40,42,44,51,52,53,54,55,56]. All studies indicated that one of the Pokémon GO benefits was improvement in the sense of connection and social interaction [27,31,32,40,49,52,54]. For example, adolescent players had better social relationships with their peers than those who did not play after 8 weeks [40]. Ewell and colleagues [27] also reported that daily time spent playing the game led to greater social interaction with both friends and strangers. However, Nikou and colleagues [64] reported contrasting findings that Pokémon GO play was not significantly associated with social integration, suggesting individuals perhaps play the game with existing friends rather than forming new relationships. Based on the findings, we speculate that although players do encounter strangers and new neighbors while playing the game, those encounters may not reach the significant magnitude, or the new relationships will not be sustained to make meaningful lasting interactions [64]. Another plausible reason for the mixed findings may be due to various samples, as individuals had different motivations for playing the game and those who played the game with the goal of increasing PA or having fun did not perceive that they had much social interaction [37]. Therefore, although the Pokémon Go game could serve as one of the platforms for better health interventions, given the relationship between sense of human connections and psychosocial and physical health, it is utterly important to consider different motivations individuals have for playing the game.

Another way Pokémon GO game improves psychological and social well-being is through reducing anxiety [32], neurotic traits [56], psychological distress [42], and improving mood [21,55]. In particular, a study [21] found the game was effective in reducing negative affect, which suggests Pokémon GO may be useful in assisting individuals with more extreme forms of negative moods. In addition, Kogan [32] concluded that playing Pokémon GO resulted in feeling less anxious about leaving the house, interacting with strangers, and going to new places. These findings are in line with mood enhancing effects of exergames [65] and findings of anxiety-reducing effects of virtual reality exercise [66]. Health practitioners, researchers, and educators can leverage these mood enhancing effects in designing effective PA interventions.

In light of cognitive influence of the game, it was found that selective attention and concentration levels in youth improved while verbal working memory improved in young adults. Cognitive wellness is crucial as it slows down the inevitable cognitive decline that comes with age. In the game, Pokémon GO players train their motor skills, reaction skills, and make strategic decisions when catching Pokémon and fighting in gym battles beyond needing to memorize Pokémon’s movement patterns during the phase they try to catch it. All of these challenges can influence mental stimulation [64]. Interestingly, some researchers did not find any significant positive impact of the game in enhancing children’s working memory [31,40], while another study [21] showed improvement in memory among young adults after the game play. The discrepancies may be due to difference in the age and exposure periods. The study with adults only lasted for one hour, while the studies with children had intervention periods of 8–10 weeks. Given that children have shorter memory spans compared to young adults [67] and that long-term high intensity PA does not lead to a beneficial development of working memory in preadolescent children (5–13 years) [68], our findings suggest Pokémon GO intervention, if used for children’s cognitive development, should be aimed to improve attention and concentration rather than working memory. However, due to paucity of research on this topic, more studies are needed in the future.

### 4.3. Limitations and Implications

This study is not without limitations. First, we did not include any qualitative studies, which may be useful in gaining perspectives on players’ game experience and motivation. Second, only three included studies implemented experimental design and none was a randomized controlled trial, making causal inferences impossible. Third, due to the lack of homogeneity in outcomes across studies, the findings should be interpreted with caution. Fourth, in studies that adopted objective measures, most used smart watches with built-in accelerometer or iPhone Health apps, where reports on validity and reliability are just emerging. Fifth, the current review is limited by the inclusion of only peer-reviewed full-text and English language publications from six databases, despite the fact other unpublished and non-English work on the same topic might be retrieved from other databases. Finally, the relatively smaller number of studies examining psychological and social outcomes limits the generalizability of the findings and, thus, more studies need to explore the implications of Pokémon GO. Future studies might be carried out in the following ways. First, future studies could also target different populations, including children and old adults with any other AR games that they may find more appealing [9]. Second, mediation effects on psychological outcomes and moderator effects of age can be investigated. For example, positive effects on psychological outcomes may be resulting from improvement in social interactions and increased PA through the game play, which warrants investigation of mediation effects [27]. Finally, the optimal intervention duration and weekly exposure can be researched to investigate the optimal length to discern significant improvement in PA, psychological and social outcomes.

Findings of this review have practical implications for health and fitness professionals, educators, and researchers. The AR game interventions can be designed for sedentary and obese populations who have low motivation and self-esteem or social deficits, to retain interest in traditional exercise regimens and facilitate them to sustain their health behaviors [69]. Well-planned cautionary measures may be essential when implementing Pokémon GO into intervention as some adverse effects of the game, such as injuries and accidents, have been raised [14]. Interventions may be implemented in clinics, community care facilities, and homes to reduce mood-related mental illnesses and support those who experience isolation and loneliness. Educators may introduce geographical significance as children play the game to propel their curiosities in their neighborhood explorations and incorporate the game into fitness classes to enhance attention and concentration, which are important cognitive capabilities that need to be developed for successful learning. The Pokémon GO industry is still in its infancy and has large quantities of potential that can serve as a creative tactic to improve physical, psychological, and social well-being.

## 5. Conclusions

This review systematically synthesized the empirical literature that explored the associations between the Pokémon GO AR mobile game, PA and psychosocial outcomes. Findings indicate that Pokémon GO was associated with increased LPA and walking, improved mood and social interaction, and some aspects of cognitive ability, including memory, attention, and concentration. However, future studies with rigorous study design, as well as validated and homogeneous outcome measures, are needed to confirm the findings and explore ways to improve the game’s current incapability for players’ long-term engagements and higher intensity PA [70]. Yet, Pokémon GO still has a promising outlook to decrease the adverse effects of the game in order to better promote the wellbeing of individuals who tend to achieve overall health in a more technological and absorbing way.

## Figures and Tables

**Table 1:** Summary of the sample, outcome, and findings of the Pokémon GO studies.

First Authors (Year)	Country	Study Design	Sample (Size, Age, Sex)	Outcomes	Instruments	Exposure	Key Findings	Effective-Ness
Alloy (2018) [21]	U.S.	Crossover experimental	59 23.33 ± 3.70 78% female	PSO: affect; empathy; working memory	Positive and negative affect schedule; interpersonal reactivity index; Alloway working memory assessment	2 conditions 1 h	The use of PG increased verbal working memory and decreased negative affect in adult population.	Y
Althoff (2016) [22]	U.S.	Longitudinal	27126 Median age = 33 25% female	PA: daily steps	Accelerometers and wrist-worn activity tracking device (Microsoft Band)	2 groups 30 days	Starting to play PG is associated with increase in PA.	Y
Barkley (2017) [23]	U.S.	Longitudinal	358 19.8 ± 2.1 52% females	PA: time spent in walking (min/day) and sedentary behavior	Int’l PA Questionnaire (IPAQ); interview; accelerometers; pedometers	1 group 3 weeks	Playing PG was associated with increased self-reported walking and decreased sedentary behavior.	Y
Beach (2019) [24]	U.S.	Cross-sectional	100 45.14 ± 17.7 52% female	PA: step counts; walked distance (miles); calories burned; time spent in sedentary behavior and different PA intensities	Omron pedometers; accelerometers	2 groups 2 h	Despite no differences in greenway walking time, PG users took fewer steps, spent more time in sedentary and light intensity activity, less time in MVPA than non- PG users.	N
Bonus (2018) [51]	U.S.	Cross-sectional	399 34.54 ± 11.37 56.6% female	PSO: social anxiety; affect; nostalgia; friendship initiation and intensification; resilience; life satisfaction; loneliness; depressions	Mini-social phobia inventory; modified differential emotions scale; time perspective inventory; brief resilience scale; satisfaction with life scale; loneliness scale; Patient health questionnaire (PHQ + 4)	2 groups	PG was associated with increased positive affect, nostalgic reverie, friendship formation, intensification, and walking. There was a significant indirect relationship between game play and depression via exercise.	Y
Broom (2018) [25]	U.K.	Longitudinal	461 28.8 ± 9.56 57% female	PA: time spent in MVPA, walking, and sitting	Adapted version of IPAQ	2 groups 3 months	PG players took more days of moderate PA and walking than non-players, but no difference in time spent in MVPA or sitting.	Y
Escaravajal-Rodríguez (2018) [26]	Spain	Cross-sectional	714 24.86 ± 5.509 38% females	PA: percentage of participants walking, time spent walking	Ad hoc opinion questionnaire	1 group	Before PG, 34.9% of the respondents did not walk at all, 25.4% walked less than 1 /day, and 30.4% walked 1–2 h/day. After PG, 44.7% of the sample walked between 1–2 h/day, and 37.1% who walked 3–4 h/day.	Y
Ewell (2020) [27]	U.S.	Longitudinal	59 26.92 ± 5.82 35% female	PSO: life satisfaction; vitality; interpersonal interaction	Satisfaction with life scale; subjective vitality scale; estimated time and number of people interacted with	1 group 7 days	Daily time spent playing PG was related to higher scores of life satisfaction, vitality, and greater social interactions.	Y
Fountaine (2018) [28]	U.S.	Cross-sectional	27 21.5 ± 2.6 70% female	PA: minutes spent in PA sedentary behavior; step counts (step/min); heart rate	Accelerometers, pedometers, heart rate monitor and watch	1 group 60 min	Playing PG for 60 min meets multiple thresholds similar to moderate-intensity PA.	Y
Gabbiadini (2018) [29]	U.S.	Cross-sectional	981 32.51 ± 10.20 62% females	(1) PA: recency and frequency of general PA; amount of PA related to PG (2) PO: attitude	(1) Questionnaire (2) Attitude regarding physical activities for health and fitness scale	1 group 1 month	PG do not lead to more PA outside of the game. The use of PG does not lead to more favorable attitude toward PA in general.	1) N 2) N
Hino (2019) [30]	Japan	Longitudinal	230 57.14 ± 9.6 50% female	PA: daily mean step counts	Omron pedometers	2 groups 9 months	The players maintained their step counts while non-players decreased their step counts in winter.	Y
Howe (2016) [49]	U.S.	Longitudinal	1182 26.49 ± 4.55 71% female	PA: changes in daily steps	Built-in accelerometers in iPhone Health application	2 groups 10 weeks	The daily average steps for PG players during the first week of installation increased by 955 additional steps, but by the sixth week, steps dropped back to pre-installation levels.	Y
Hsien (2019) [31]	Taiwan	Experimental	123 12.58 ± 1.03 51.2% female	PSO: memory; selective attention; cognitive performance; creativity emotional intelligence; sociability	Wechsler memory scale-Chinese revision test; attention for children of elementary school; test of creative imagination; Trait and emotional intelligence questionnaire	2 groups 10 weeks	PG showed a significant increase in their selective attention, concentration levels, creative imagination, emotionality, and sociability levels.	Y
Kogan (2017) [32]	U.S.	Cross-sectional	269 18 and older 68% female	(1) PA: walking, running, biking and skating (hours/day) (2) PSO: time spent for social interaction; Anxiety	(1,2) Questionnaires	1 group	A significant increase in all types of exercise was found between pre-and post-PG. A significant number of players reported spending more time with family members and feeling less anxious after PG.	(1) Y (2) Y
Langford (2019) [33]	U.S.	Cross-sectional	20 26.9 ± 5.38 55% female	PA: daily energy expenditure; time spent in different PA intensity	Sense wear armband (SWA)	2 groups 3 months	Hours spent in light and moderate PA was not significantly different between the groups.	N
Liu (2017) [34]	U.S.	Cross-sectional	47 28.7 ± 5.9 47% female	PA: exercise frequency (days per week) and duration (minutes), daily steps	Questionnaire	1 group	These participants reported playing PG added 2.8 h/week of PA participation and 5.6 mile/week of distance travelled.	Y
Ma (2018) [35]	Hong Kong	Longitudinal	210 26.1 ± 8.7 (range 13–65) 33.8% female	PA: daily walking and running distances (km)	Mobile phone	1 group 21 days	PG was associated with a short-term increase in the players’ daily walking and running distances.	Y
Madrigal Pana (2019) [36]	Costa Rica	Cross-sectional	1059 18+ 52% female	PA: walking distance (km); steps	National household survey	1 group	Current PG players (*n* = 24) reported spending 6.7 h per week playing the game and walking 24.7 km.	Y
Marquet (2018) [37]	U.S.	Cross-sectional	74 19.6 50% female	PA: daily steps count	Step counting app; IPAQ short form; ecological momentary assessment app	2 groups 7 days	PG was associated with higher number of steps compared to non-playing day.	Y
Militello (2018) [38]	U.S.	Retrospective cohort	160 adults 18+ 71.9% female; 31 children 5–17 y 28.8% girls	PA: strenuous, moderate, mild PA (minutes/week; minutes/day)	Revised Godin leisure time exercise questionnaires (r-GLTEQ)	1 group	For parents, there were significant increases in minutes spent in mild and moderate PA per week after playing PG.	Y
Ni (2019) [48]	Hong Kong	Longitudinal	65 20.7 (IQR 19–22) 66% female	PA: change in daily walking distance (km/day)	iPhone Health application; questionnaires	2 groups 50 days	PG players on average walked more daily on the first week; however, no differences were seen in the following weeks.	Y
Nigg (2018) [50]	U.S.	Retrospective cohort	486 28.6 ± 8.5 57.8% female	PA: strenuous, moderate, mild PA (minutes/day); sedentary time (minutes/day)	r-GLTEQ	1 group	Playing PG increased MVPA by about 50 min per week and reduced sedentary behavior by about 30 min per day.	Y
Purda (2019) [39]	Serbia	Experimental	32 18–49 years 62.5% female	PA: walking distance (km); steps	Pedometer—step counter free and calorie burner	3 groups 5 week	On average regular trainer group walked the most, while new trainer group walked less than regular trainer group, but more than non-trainer group.	Y
Ruiz Ariza (2018) [40]	Spain	Longitudinal	190 13.32 ±1.07 49% female	(1) PA: MVPA (day/week); walking (minutes/day, miles) (2) PSO: memory; selective attention; concentration; math and reading ability; emotional intelligence; sociability	(1) Adolescent PA measure questionnaire (2) d2 test; Neale analysis of reading ability; Reynolds intellectual assessment scale; Trait and emotional intelligence questionnaire short form	2 groups 8 weeks	Players walked 54 km and spent 40 min/day playing. PG increases the amount of daily exercise in adolescents. PG players significantly increased their selective attention, concentration levels, and sociability levels against their peers.	(1) Y (2) Y
Schade (2020) [41]	U.S.	Cross-sectional	27 21.1 ± 3.4 48% female	PA: daily step counts distance travelled(km)	Fitbit charge heart rate FB405BKL monitors	2 groups 2 weeks	No significant difference was found between the two groups on daily steps and distance.	N
Watanabe (2017) [42]	Japan	Longitudinal	2530 42.12 (10.79) 37.5% female	PO: psychological distress, physical complaints, job performance	Questionnaires; brief job stress questionnaire; WHO health and work performance questionnaire	2 groups 1 month or longer	Improvement in psychological distress was significantly greater among PG players than among non-players.	Y
Wattanapisit (2018) [43]	Thailand	Longitudinal	26 22.04 ± 1.70 26.9% female	PA: time spent in sedentary behavior and PA	Self-administered questionnaire	1 group 3 months	There was no statistically significant change in PA.	N
Williams (2019) [44]	U.S.	Cross-sectional	438 18–60+	PSO: social life change; mood and sense of success after game play	Questionnaires	1 group 1–5 months	There is a strong correlation between PA and the feeling of happiness	Y
Wong (2017) [45]	Hong Kong	Cross-sectional	644 18–60 52% female	PA: MVPA, walking and total PA (MET—min/week)	IPAQ-short form;	3 groups 28 days	There was no significant difference in PA levels between the three groups.	N
Xian (2017) [46]	U.S.	Longitudinal	167 Median 25 (IQR 21–29) 48% female	PA: daily average step count; percentage of days achieving more than 10,000 steps	iPhone Health application; questionnaires	1 group 3 weeks	The percentage of days with >10 000 steps per day increased from 15.3% before to 27.5% after playing PG (a 12.2% increase).	Y
Yan (2020) [47]	U.S.	Cross-sectional	393 19.03 ± 2.04 44.5% female	PA: time spent in walking, jogging, and total PA participation	Questionnaires	3 groups 7 days	The non-users spent significantly more time walking than non-active users and active users.	N
Evans (2019) [52]	U.K	Cross-sectional	375 56.8% female	(1) PA: daily movements (2) SO: sociability	(1,2) Questionnaires	1 group	79% and 49.6% of participants reported PG had impacted their daily movements and led them to making new friends, respectively.	(1) Y (2) Y
Kaczmarek (2017) [53]	Poland	Longitudinal	444 23.4 ± 5.88 49.3/% female	PA levels	IPAQ	1 group 16–90 days	Individuals who spent more time playing PG at Time 1 (T1) were more physically active and spent more time outdoors at 6 weeks after (T2).	Y
Kim (2020) [54]	U.S.	Cross-sectional	325 19.2 ± 2.55 55% female	SO: feeling of presence and sense of community belonging	Questionnaires	1 group	A feeling of presence while playing PG was positively associated with a sense of community belonging and exploring the community.	Y
Zach (2017) [55]	U.S	Cross-sectional	405 50% female	PO: enjoyment; game play behavior; behavioral consequences; psychosocial wellbeing	Questionnaires	1 group	38% of respondents reported daily game playing, and 20% reported playing for at least 2 h on any given day. Factor analysis showed that participants experienced improvement in cognition, emotion, and communication.	Y
Mattheiss (2017) [56]	Austria	Longitudinal	335 29.5 ± 9.05 53.7% female	PO: personality	Big Five	2 groups 3 months	Players who continued playing three months later had a lower score in “Neuroticism” than those who stopped playing.	Y

Note: PG, Pokémon GO; PA, physical activity; PSO, psychological and social outcomes; PO, psychological outcomes; SO, social outcomes; Y, yes; N, no; MVPA, moderate-to-vigorous physical activity.

## Data Availability

The data presented in this study are available in Table 1 and Appendix A Table A1.

## Appendix A

**Table A1:** Design quality analysis for the Pokémon GO studies.

Study ID/Component Ratings	40	47	42	51	23	49	48	21	31	24	25	26	33	50	39	41	55	56
**Selection bias**																		
Q1: Are the individuals selected to participate in the study likely to be representative of the target population? ^a^	3	2	2	1	2	1	3	3	2	2	2	2	2	2	4	2	2	5
Q2: What percentage of selected individuals agreed to participate? ^b^	2	5	5	5	5	5	1	5	1	5	5	5	5	5	5	5	2	5
**RATE THIS SECTION ^c^**	3	2	2	2	2	2	3	3	2	2	2	2	2	2	3	2	2	2
**Study design**																		
Q1: Indicate the study design ^d^	3	7	6	7	5	3	3	2	2	7	3	7	7	5	2	7	7	6
**RATE THIS SECTION ^c^**	2	3	2	3	2	2	2	1	1	3	2	3	3	2	1	3	3	2
**Confounders**																		
Q1: Were there important differences between groups prior to the intervention? ^e^	1	2	1	3		2	1		1	1	1		1		3	2		3
Q2: If yes, indicate the percentage of relevant confounders that were controlled (either in the design (e.g., stratification, matching) or analysis)? ^f^	1	N/A	1	4		N/A	1		1	1	3		1		4	N/A		4
**RATE THIS SECTION ^c^**	1	1	1	3	N/A	1	1	N/A	1	1	3	N/A	1	N/A	3	1	N/A	3
**Blinding**																		
Q1: Was the outcome assessor(s) aware of the intervention or exposure status of participants? ^g^	1	1	1	1	1	1	1	1	1	1	1	1	1	1	1	1	1	1
Q2: Were the study participants aware of the research question? ^h^	1	1	1	1	1	1	1	1	3	1	1	1	1	1	1	2	1	2
**RATE THIS SECTION ^c^**	3	3	3	3	3	3	3	3	2	3	3	3	3	3	3	2	3	2
**Data collection methods**																		
Q1: Were data collection tools shown to be valid? ^i^	1	3	1	3	1	1	1	1	1	1	1	3	1	1	3	1	3	1
Q2: Were data collection tools shown to be reliable? ^j^	1	1	1	3	1	1	1	1	1	1	1	3	3	1	3	1	3	1
**RATE THIS SECTION ^c^**	1	2	1	3	1	1	1	1	1	1	1	3	2	1	3	1	3	1
**Withdrawal and dropouts**																		
Q1: Were withdrawals and dropouts reported in terms of numbers and/or reasons per group? ^k^	2	2	1	4	2	2	1	2	1	4	2	4	2	4	2	2	4	1
Q2: Indicate the percentage of participants completing the study. (If the percentage differs by groups, record the lowest) ^l^	1	4	2	5	5	1	1	4	1	5	3	5	5	5	5	5	5	3
**RATE THIS SECTION ^c^**	1	3	2	3	2	1	1	3	1	2	3	2	2	2	2	2	2	3
**Global rating ^m^**																		
**RATE THIS SECTION ^c^**	3	3	2	3	2	2	3	3	1	3	3	3	3	2	3	2	3	3
**Study ID/ Component Ratings**	**22**	**28**	**37**	**46**	**30**	**35**	**38**	**42**	**27**	**44**	**29**	**45**	**34**	**32**	**36**	**52**	**53**	**54**
**Selection bias**																		
Q1: Are the individuals selected to participate in the study likely to be representative of the target population? ^a^	2	3	2	2	1	2	2	2	2	2	2	2	2	3	1	2	2	2
Q2: What percentage of selected individuals agreed to participate? ^b^	1	5	5	5	2	1	5	3	2	1	5	3	5	5	5	5	5	5
**RATE THIS SECTION ^c^**	2	3	2	2	2	2	2	3	2	2	2	3	2	3	2	2	2	2
**Study design**																		
Q1: Indicate the study design ^d^	3	7	7	5	6	5	5	5	6	7	7	7	7	7	7	7	6	7
**RATE THIS SECTION ^c^**	2	3	3	2	2	2	2	2	2	3	3	3	3	3	3	3	2	3
**Confounders**																		
Q1: Were there important differences between groups prior to the intervention? ^e^	2				2							3						
Q2: If yes, indicate the percentage of relevant confounders that were controlled (either in the design (e.g., stratification, matching) or analysis)? ^f^	N/A				N/A							4						
**RATE THIS SECTION ^c^**	1	N/A	N/A	N/A	1	N/A	N/A	N/A	N/A	N/A	N/A	3	N/A	N/A	N/A	N/A	N/A	N/A
**Blinding**																		
Q1: Was the outcome assessor(s) aware of the intervention or exposure status of participants? ^g^	1	1	1	1	1	1	1	1	1	1	1	1	1	1	1	1	1	1
Q2: Were the study participants aware of the research question? ^h^	3	1	1	1	2	1	1	1	1	1	1	1	1	1	1	1	2	2
**RATE THIS SECTION ^c^**	2	3	1	3	2	3	3	3	3	3	3	3	3	3	3	3	2	2
**Data collection methods**																		
Q1: Were data collection tools shown to be valid? ^i^	1	1	1	1	1	1	1	1	1	1	3	1	1	1	3	3	1	1
Q2: Were data collection tools shown to be reliable? ^j^	1	1	1	1	1	1	1	3	1	1	1	1	1	1	3	3	1	1
**RATE THIS SECTION ^c^**	1	1	1	1	1	1	1	2	1	1	2	1	1	1	3	3	1	1
**Withdrawal and dropouts**																		
Q1: Were withdrawals and dropouts reported in terms of numbers and/or reasons per group? ^k^	2	1	2	2	4	2	4	1	1	2	2	2	4	4	4	4	1	4
Q2: Indicate the percentage of participants completing the study. (If the percentage differs by groups, record the lowest) ^l^	4	1	1	4	5	4	5	3	1	3	4	1	5	1	5	5	3	5
**RATE THIS SECTION ^c^**	3	1	1	3	2	3	2	3	1	3	3	1	3	1	3	2	3	2
**Global rating ^m^**																		
**RATE THIS SECTION ^c^**	2	3	2	3	1	3	2	3	2	3	3	3	3	3	3	3	2	2

^a^ Categories for selection bias Q1: 1. Very likely; 2. Somewhat Likely; 3. Not likely; 4. Cannot tell. ^b^ Categories for selection bias Q2: 1. 80%–100% agreement; 2. 60%–79%; 3. Less than 60%; 4. Not applicable; 5. Cannot tell. ^c^ Categories for section rating: 1. Strong; 2. Moderate; 3. Weak. ^d^ Categories for study design: 1-Randomized controlled trial; 2-Controlled clinical trial; 3-Cohort analytic; 4-Case-control; 5-Cohort; 6-Interrupted time series; 7-Other specify-cross-sectional. ^e^ Categories for confounders Q1: 1. Yes; 2. No; 3. Cannot tell. ^f^ Categories for confounders Q2: 1. 80%–100%; 2. 60%–79%; 3. Less than 60%; 4. Cannot tell. ^g^ Categories for blinding Q1: 1. Yes; 2. No; 3. Cannot tell. ^h^ Categories for blinding Q2: 1. Yes; 2. No; 3. Cannot tell. ^I^ Categories for data collection methods Q1: 1. Yes; 2. No; 3. Cannot tell. ^j^ Categories for data collection methods Q2: 1. Yes; 2. No; 3. Cannot tell. ^k^ Categories for withdrawals and dropouts Q1: 1. Yes; 2. No; 3. Cannot tell; 4. Not applicable. ^l^ Categories for withdrawals and dropouts Q2: 1. 80%–100%; 2. 60%–79%; 3. Less than 60%; 4. Cannot tell; 5. Not applicable. ^m^ Categories for global rating: 1. Strong (no WEAK ratings); 2. Moderate (one WEAK rating; 3. Weak (two or more WEAK rating).
